# Configuring Therapeutic Aspects of Immune Checkpoints in Lung Cancer

**DOI:** 10.3390/cancers15020543

**Published:** 2023-01-16

**Authors:** Avinash Khadela, Vivek P. Chavda, Humzah Postwala, Ramya Ephraim, Vasso Apostolopoulos, Yesha Shah

**Affiliations:** 1 Department of Pharmacology, L. M. College of Pharmacy, Navrangpura, Ahmedabad 380009, Gujarat, India; 2 Department of Pharmaceutics and Pharmaceutical Technology, L. M. College of Pharmacy, Navrangpura, Ahmedabad 380009, Gujarat, India; 3 Pharm. D Section, L. M. College of Pharmacy, Navrangpura, Ahmedabad 380009, Gujarat, India; 4 Institute for Health and Sport, Victoria University, Melbourne, VIC 3030, Australia; 5 Australian Institute for Musculoskeletal Science, Melbourne, VIC 3021, Australia

**Keywords:** immune checkpoint inhibitors, lung cancer, PD-1/PD-L1, CTLA-4, immune editing, programmed cell death protein, programmed cell death ligand

## Abstract

**Simple Summary:**

There is a need to improve the conventional treatment options for lung cancer. Immunotherapy is based on the premise that therapeutic drugs destroy tumor cells by stimulating the immune response. Drugs targeting immune checkpoints belong to the class of immunotherapy. These are specific antibodies targeted against immune checkpoints called immune checkpoint inhibitors. Herein, we focus on the agents targeting these checkpoints as well as exploring novel checkpoints that can be prospectively targeted.

**Abstract:**

Immune checkpoints are unique components of the body’s defense mechanism that safeguard the body from immune responses that are potent enough to harm healthy body cells. When proteins present on the surface of T cells recognize and bind to the proteins present on other tumor cells, immune checkpoints are triggered. These proteins are called immunological checkpoints. The T cells receive an on/off signal when the checkpoints interact with companion proteins. This might avert the host’s immune system from eliminating cancer cells. The standard care plan for the treatment of non-small cell lung cancer (NSCLC) has been revolutionized with the use of drugs targeting immune checkpoints, in particular programmed cell death protein 1. These drugs are now extended for their potential to manage SCLC. However, it is acknowledged that these drugs have specific immune related adverse effects. Herein, we discuss the use of immune checkpoint inhibitors in patients with NSCLC and SCLC, their outcomes, and future perspectives.

## 1. Introduction

Lung cancer is difficult to treat and in 2020 it accounted for 11.4% of new cancer cases and 18% of cancer-related deaths globally [1]. In the United States alone there were 1.8 million new cancer cases and 608,570 cancer deaths in 2021 [2]. Lung cancer has the highest rate of associated mortality and is the third most commonly occurring cancer worldwide [3]. The 5-year survival rates for non-small cell lung cancer (NSCLC) and SCLC are quite low, at 6% and 7, respectively [4]. The four pillars of management in lung cancer are surgery, radiotherapy, chemotherapy, and palliative care [5]. Adjuvant or neoadjuvant chemotherapy and radiotherapy remain the gold standard as first-line management strategies and in most cases the overall survival rate is extended by 6 months [6]. As a result, they have not produced the intended clinical results and are linked to a high rate of recurrence and poor prognosis [7]. Immunotherapy is a subcategory of cancer management strategy that works by inducing the production of various anti-tumor molecules in the plasma including antibodies (Abs) and other immune-mediated cells [8]. Immunotherapeutic drugs induce tumor cell apoptosis following their interaction with the immune system [9]. Owing to the ineffectiveness of immunotherapy in the management of lung cancer, it is considered to be non-immunogenic [10].

Understanding the biological and clinical features of lung cancer is crucial to test various immune checkpoint inhibitors (ICIs) for its management [11]. The number of genetic changes, or the tumor mutational burden, is high in lung cancer, making it more immunogenic and susceptible to ICI-response. These ICIs are mainly responsible for the reduction of antigen-specific immune reactions by acting as coinhibitory factors [12]. The prototypical ICIs for lung cancer, also known as programmed death protein 1 (PD-1), and its ligand, programmed cell death ligand 1 (PD-L1) and cytotoxic T-lymphocyte-associated antigen 4 (CTLA-4), are the most studied ICI targets. The T cell surface expresses CTLA-4, which contributes to the initial activation of T-cells in the lymph nodes. Subsequently, it binds to its receptors CD80/CD86 present on antigen-presenting cells and provides inhibitory signals to T cells [13]. T cells are inhibited when PD-1 present on T cells interacts with PD-L1 expressed on antigen presenting cells. The inhibition of these checkpoints leads to the activation of T cell-mediated immunity and anti-cancer effects [14]. Currently, nivolumab, pembrolizumab, atezolizumab, durvalumab, and cemiplimab have been approved for NSCLC, and for SCLC durvalumab is approved. Apart from PD-1 and CTLA-4, various other molecules on these targets, as well as newer ICI targets, are being identified. These include the human endogenous retrovirus-H long terminal repeat-associating protein 2 (HHLA-2), the lymphocyte activation gene 3 (LAG-3), the V-domain immunoglobulin-containing suppressor of T cell activation (VISTA), the T cell immunoglobulin and mucin domain 3 (TIM-3) and the T cell immunoreceptor with Ig and immunoreceptor tyrosine-based inhibitory motif domains (TIGIT) [15]. Herein we discuss the importance of ICIs in the management of NSCLC/SCLC and the role of immunological checkpoints in cancer progression.

## 2. Significance of Immune Checkpoint Inhibitors

Most lung cancer patients are diagnosed at quite advanced stages, to which chemotherapy has shown minimal success, treating only 15–30% of all lung cancer cases [16]. Immunotherapy can be utilized in the early stages of lung cancer. Certain immune cells are involved in the control and/or destruction of the tumor cells including T-cells, natural killer (NK) cells, dendritic cells (DC), antigen presenting cells, and various cytokines [17]. However, interleukin (IL)-12 secreted by antigen presenting cells activates CD8+ T cells, which direct the destruction of tumor cells [18]. Concomitantly, CD4+ T helper (Th)-1 cells activate CD8+ T cells by secreting interferon (IFN)-γ and CD4+ Th2 cells elicit an antibody-mediated reaction by activating B-cells through IL-4 [8].

### 2.1. Immunoediting

Tumor cells have developed mechanisms to bypass the immune system of the host, i.e., immunoediting. Based on the extrinsic tumor microenvironment (TME), tumor cells alter their immunogenicity in three phases: (1) elimination, (2) equilibrium and (3) escape [19]. Initially, tumor cells are destroyed by the immune cells in the TME mediated by innate and adaptive immunity before they become clinically significant via instigating apoptosis. However, some of these tumor cells survive the initial elimination phase, after which they enter into the equilibrium phase [20]. The elimination phase is a dormant phase that can span over years. During this phase, the immunity of the body becomes compromised significantly. In this situation, the immune cells’ primary function is to stop the growth of tumor cells. When tumor cells with low immunogenicity multiply, their dormancy in the equilibrium phase is abruptly broken, signaling their entry into the final escape phase. Tumor cells grow by passing various regulatory pathways, apoptosis, and by establishing an immune suppressive TME [21,22,23]. These cells continue to grow and mimic the action of the surrounding cells and become clinically significant by this stage. The angiogenesis and immune microenvironment are two closely related branches of TME and eventually have become very important approaches for the development of newer therapies [24]. In addition, chemokines are involved in the progression of cancer, and immunotherapy’s targeting of such chemokines can aid in the development of new targets [20,25]. The epithelial–mesenchymal transition (EMT) also plays an important role in driving metastasis and modulating the immune response. Moreover, EMT is also associated with the increased expression of ICIs such as CTLA-4, TIM-3 and cytokines such as IL-10 and TGF-β [26]. Thus, EMT plays a clear role in suppressing immune responses. Hence, targeting EMT could provide newer opportunities for immunotherapy [27]. Similarly, macrophages constitute the major portion of the immune cells and these pro-tumorigenic macrophages interfere with the local TME and result in cancer growth and proliferation [28]. Some recent clinical studies have also shown the great potential of macrophages in improving the approaches for immunotherapy.

### 2.2. Tumor Mutational Burden

There are large number of mutations seen in cancer patients which tell us about the environmental factors and biological processes responsible for the gene mutation, as xenobiotics and endogenous changes are responsible for alterations in the DNA [29]. The number of somatic mutations per mega-base pair of interrogated genomic sequences is referred to as tumor mutational burden (TMB). TMB is basically the reflection of the number of tumor specific reflections occurring in the tumor. These newer mutations create neo-epitopes which can be targeted by the immune system and especially by immune checkpoint inhibitions [29]. TMB is an index used to estimate the mutations harbored by cancer cells and can be estimated using various sequencing techniques such as next-generation sequencing, whole genome, and whole exome sequencing [30]. To estimate the TMB, whole exome sequencing is regarded as the gold standard. Lung cancer is associated with having the highest TMB, probably due to the higher presence of mutagens while smoking. Several studies have shown an association between smoking status and high TMB [30]. Subgroup analyses demonstrated that nivolumab and other immune therapies are highly efficacious in NSCLC patients with high TMB [30]. Accordingly, nivolumab and ipilimumab show superior outcomes in terms of enhanced progression-free survival and objective response rate in patients with SCLC [31]. The results are supported by the recent checkmate 227 study, which noted that subjects with TMB (>10 mu/mb) have improved response rates following ipilimumab treatment as a first-line drug instead of chemotherapy for NSCLC [32,33]. Thus, immunotherapy can produce better results in patients with high TMB.

## 3. Mechanism of Action of ICIs

**Figure 1:**
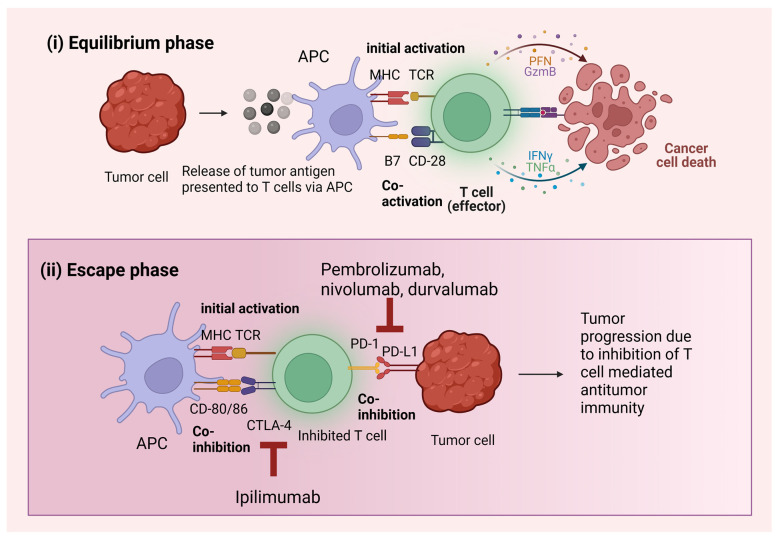
Tumor behavior in the presence and absence of ICIs. (**i**) Tumor cells in the equilibrium phase do not survive due to the presence of initial as well as co-activation markers. (**ii**) Tumor progresses due to the absence of co-activation. Initial activation is present along with co-inhibition mediated by immune checkpoints. The ICIs are antibodies targeting these immune checkpoints, such as pembrolizumab, nivolumab, durvalumab, and ipilimumab. Abbreviations: APC: antigen presenting cells, MHC: major histocompatibility complex, TCR: T cell receptor, CD: cluster of differentiation, PFN: pore forming granule protein perforin, GzmB: granzyme B, IFN: interferon, TNF: tumor necrosis factor, CTLA: cytotoxic T lymphocyte associated antigen, PD-1: programed cell death receptor, PD-L1: programed cell death ligand.

The host immune system, under normal circumstances, is activated and undergoes a cascade of immune reactions against foreign cells. The immune checkpoints exist to keep these activated immune cells in check so that there are no overshot immune reactions. Therefore, once an immune response is triggered, these checkpoints function as inhibitory receptors to prevent further immune cell activation [34]. When a tumor is formed, cancer immunity cycles are activated in which antigen presentation takes place to activate T cells leading to their migration and infiltration into the TME [35]. Tumor cells have two phases in their cycle: (i) the equilibrium phase and (ii) the escape phase. In the equilibrium phase, the cells are not immunogenic enough to present cancer antigens to initiate the cancer immunity cycle. The TMB of the tumor cells rises during the escape phase, which causes the activation of several immunosuppressive systems, including immunological checkpoints. Most tumors are detectable only after reaching the escape phase [36] (Figure 1). In the presence of a tumor, the early inhibitory signal from the checkpoints does not allow the immune cells such as T cells to exert complete immune destruction of the non-self-cancerous cells. By inhibiting the signals of the immune checkpoints, ICIs aid immune cells in exerting their anti-tumor immunity against tumor cells [37].

ICIs are antibodies that target immunological checkpoints that are important for the growth of tumors by preventing T cell-mediated defense against cancer [38]. These ICIs have been explored as working on various targets. Mainly, they are efficacious in tumors as the host immune system recognizes them as non-self. In other words, those tumors having the highest TMB are generally more sensitive to the activity of these ICIs [39]. In current clinical practice, the most commonly targeted checkpoints by ICIs are PD-1 and CTLA-4. These checkpoints block the immune response against tumors by manipulating T cells [40]. These, as well as other checkpoints, can be targeted in the treatment of lung cancer.

### 3.1. PD-1 Pathway

PD-1, a transmembrane coinhibitory receptor, is expressed on the surface of T cells after activation, and its ligand PD-L1/2 is generally expressed by the tumor cells [41,42]. PDL-1 expression is generally regulated by Janus Kinase/Signal transducer and activator of transcriptor (JAK/STAT) via interferon regulating factors (IRF)-1, whereas PDL-2 is regulated by IFN-γ/β. Th cells induce the production of these ligands [43,44,45]. A study was carried out in which the *Pdcd1* gene responsible for encoding PD-1 was knocked out in mice. This genetic loss potentiated autoimmunity as seen with the development of lupus-like disease. This shows the role of PD-1 as an immune checkpoint involved in the pathogenesis of lung cancer [46]. The immunoreceptor tyrosine-based inhibitory motif (ITIM) and immunoreceptor tyrosine-based switch motif (ITSM) are phosphorylated upon binding PD-1 present on activated peripheral T cells to its ligands PD-L1 or PD-L2 presented by the antigen presenting cells, which recruit various tyrosine phosphates such as Src homology 2 domain-containing phosphatases (SHP) 1 and 2. Out of these, SHP2 is mainly responsible for PD-1-mediated inhibitory functions [47]. This binding subsequently downregulates T cell-mediated tumor cell destruction via major histocompatibility complex (MHC) mediated tumor antigen presentation [48]. The major reason for this downregulation is the CD28-mediated inhibition of various signaling pathways, including phosphoinositide-3 kinase Ak strain transforming (PI3K/AKT) and Rat sarcoma virus/mitogen-activated protein kinase/extracellular signal-regulated kinase (RAS/MEK/ERK) [49]. To bypass this downregulation of anti-tumor immune response, antibodies against PD-1/PD-L1 are administered which prevent the interaction between PD-1 and PD-L1. Microsatellite instability and PD-1 expression can be employed as prognostic indicators to forecast the performance of PD-1/PD-L1 targeted ICIs [50]. These ICIs mainly act on the stage of the cancer immunity cycle where activated T cells recognize and eliminate tumor cells [49].

### 3.2. CTLA-4 Pathway

The co-inhibitory receptor CTLA-4, also known as CD152, is a CD28 homolog expressed on activated regulatory T cells (Tregs) and functions as an immunological checkpoint [51]. In humans, lung cancer cell lines exposed to CTLA-4 antibody showed anti-cancer effects, thus becoming a prospect for further research [52]. Antigen presenting cells express ligands CD80/86 for further binding to CTLA-4. The CD4+ and CD8+ T-cells that have been activated also express CTLA-4 which binds to the same ligands [53]. However, CTLA-4 binds to CD80/86 with a higher affinity compared to CD28 [54]. The interaction of CTLA-4 to its ligands stimulates the coinhibitory signals on T cells exerting anti-tumor immune reaction, thus downregulating the immune response [43]. The interaction of the T cell receptors with the MHC-cancer antigen complex presented by antigen presenting cells, together with co-stimulation by CD80/86, is necessary for the activation of T cells. During the overexpression of CTLA-4, this costimulatory signal is blocked which inhibits the response mediated by T cells. [55]. The suppression of cellular immunological responses mediated by CTLA-4 involves both intrinsic and extrinsic pathways. The inhibition of protein translation, phosphatase recruitment, and cytokine signaling inhibition are the processes that take place in the intrinsic pathway, Whereas in the extrinsic pathway CD28 competes with CD80/86 for binding, which is eliminated. Suppressive indoleamine (2,3)-dioxygenase (IDO) is released, and Treg activity is modulated [56,57]. CTLA-4 on activation is known to phosphorylate and binds to SHP2 and PP2A [58]. CTLA-4 inhibitors are ICI antibodies against CTLA-4 that act by binding to CTLA-4 and prevent interaction with its ligand that subsequently inhibits the immune downregulatory response of CTLA-4. Unlike anti-PD-1 antibodies, the antibodies against CTLA-4 act at a stage where T cells are activated after being presented with the cancer antigens via antigen presenting cells [59]. These antibodies block the CTLA-4 binding, thus CD28 binds to its ligand CD80/86 despite having a lower affinity than CTLA-4. Apart from subsequent T cell activation, CTLA-4 present on the surface of Tregs is also inhibited by these ICIs, thus eliminating them [60].

### 3.3. Other Immune Checkpoints

**Figure 2:**
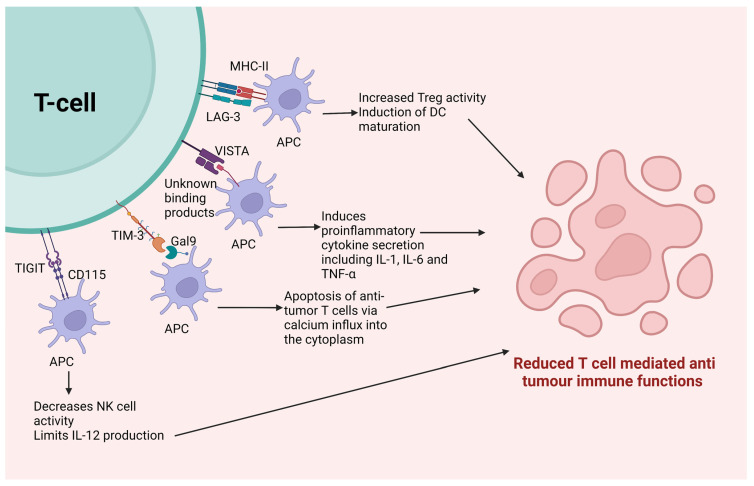
Immune checkpoints other than PD-1 and CTLA-4. Various immune checkpoints that are expressed on T cells following binding to their particular ligands lead to a series of immunosuppressive reactions that ultimately suppress the anti-tumor immune activity of functional T cells. Abbreviations: APC: antigen presenting cells, CD: cluster of differentiation, DC: dendritic cells, Gal9: Galectin-9, IL: interleukin, MHC: major histocompatibility complex, LAG-3: Lymphocyte activation gene-3, TIGIT: T Cell Ig and Immunoreceptor Tyrosine-Based Inhibitory Motif Doman, TIM-3: T Cell Immunoglobulin and Mucin-Containing Protein 3, VISTA: V-Domain Ig Suppressor of T Cell Activation.

The clinical results of currently used ICIs, though impressive, show inefficacy in some patients owing to the feedback or compensatory inhibition of the T cell signaling pathway [53]. Thus, apart from the PD-1 and CTLA-4 prototypic ICIs, several other alternative immune checkpoints can be targeted with antibodies. These can be used along with the currently used ICIs to provide enhanced results by targeting multiple targets [61]. Unlike the previously mentioned two immune checkpoints, the following checkpoints do not have a conclusive role in the development or progression of NSCLC or SCLC. They are being explored as prospective checkpoints that might be able to improve disease outcomes for lung cancer. The mechanism of these ICIs has been graphically described in Figure 2.

#### 3.3.1. Lymphocyte Activation Gene-3 (LAG-3)

Lymphocyte activation gene-3 (LAG-3) or CD223, an immune checkpoint, shows homology with CD4. Apart from activated T cells and Tregs, LAG-3 is mostly expressed on tumor-infiltrating lymphocytes (TILs) and NK cells. [62]. It has a significantly stronger affinity for its ligand MHC-II than CD4 does [63]. LAG-3 is expressed on various cells depending on the inflammatory cytokine levels, such as interleukin (IL)-2/7/12 and IFN-γ [64]. This engages in an interaction with the MHC-II that is expressed by antigen presenting cells, tumor cells, and cancer-associated fibroblasts (CAFs), which reduce T cell activation and immunological responses while increasing Treg activity [65]. On activation of LAG-3, it is known to express a specific KIEELE motif that is responsible for inhibiting T-cell activity [62]. Apart from this, the induction of DC maturation takes place owing to the interaction of LAG-3 and its ligand MHC-II. There is a strong correlation between the expression of LAG-3 and PD-1 expression in various solid tumors, including NSCLC. In vivo murine models have shown the synergy between these checkpoints [66]. Targeting both PD-1 and LAG-3 simultaneously using ICIs proved to be efficacious in tumors that were resistant to the action of any of these ICIs used as monotherapy [67]. The LAG-3-targeting ICIs can suppress Tregs while activating effector T cells. Several agents are under evaluation in human phase I/II clinical studies for a variety of solid malignancies, given in different combinations [68]. Relatlimab is one such FDA approved agent targeting LAG-3 for advanced melanoma [69].

#### 3.3.2. V-Domain Ig Suppressor of T Cell Activation (VISTA)

V-Domain Ig Suppressor of T Cell Activation (VISTA) is structurally homologous to PD-L1. VISTA has been illustrated to act on both ligands, as well as the receptors for APCs and T cells, respectively [70]. The primary distinction between VISTA and other checkpoints is that the former is expressed on naïve T cells, as opposed to the latter, which are largely expressed on T cells that have already been activated [71]. It has unknown binding partners and is mainly expressed in hematopoietic (myeloid) cells, DCs, macrophages, and monocytes. When activated, VISTA leads to the dampened activity of tumor-specific T cells and includes cytokine secretion [72]. Post-ipilimumab therapy in prostate cancer patients, VISTA levels are upregulated, suggesting its role in ICI-resistance [73]. Thus, anti-VISTA antibodies can be used along with other ICIs in a non-overlapping fashion to give much higher efficacy [74]. Mice with deleted VISTA gene show stronger responses when exposed to antigens [71]. In vivo studies have shown delayed tumor growth when mice are administered with anti-VISTA antibodies by enhancing the function of T cell-mediated anti-tumor immunity. In addition, these agents are known to have impressive efficacy despite the absence of epidermal growth factor receptor (EGFR) mutations. or in tumors with low TMB where other ICIs fail to prove their efficacy [75]. CA-170, an oral drug, is an example of dual checkpoint targeting that acts by inhibiting both VISTA and PD-L1, increasing clinical benefit by 75%, and progression free survival to 19.5 weeks in NSCLC patients. Apart from this, various other ICIs targeting VISTA are being evaluated in different stages of clinical trials (Table 1) [61].

#### 3.3.3. Human Endogenous Retrovirus-H Long Terminal Repeat-Associating Protein 2 (HHLA2)

Human endogenous retrovirus-H long terminal repeat-associating protein 2 (HHLA2) is a recently discovered immune checkpoint homologous to the B7 family [76]. It is mostly expressed on the human monocyte’s surface and its activation is triggered by B cells [77]. The ligand to which it interacts is the transmembrane and immunoglobulin domain containing 2 (TIMGD2). When it binds to this ligand, a co-stimulatory signal for T cell activation is triggered along with the release of cytokines including IFN-γ, tumor necrosis factor-alpha (TNF-α) and IL-5/IL-10. The function of HHLA2 was implicated in lung cancer in vitro following knockdown studies in lung cancer cell lines which resulted in decreased cancer cell proliferation [78,79]. The targets against this checkpoint are still in the preclinical phase and much research is required before establishing the efficacy of ICIs against HHLA2 in humans [80].

#### 3.3.4. T Cell Immunoglobulin and Mucin-Containing Protein 3 (TIM-3)

T cell Immunoglobulin and mucin-containing protein 3 (TIM-3) receptors are specifically found on IFN-γ producing CD4+ Th cells, CD8+ T cells, Tregs and NK cells [81]. Almost 30% of CD8+ and 60% of Treg cells in NSCLC are known to express TIM-3 [82]. These receptors bind to their ligand, the C-type lectin Galectin-9 (Gal-9), which is highly expressed in tumor cells. When the receptor on CD4 Th cells binds to this ligand, it leads to apoptosis of T cells via induction of calcium influx into the cytoplasm [83]. When the ligand interacts with TIM-3 expressed on CD8+ T cells, the receptor colocalizes with CD45 and CD148 [84]. This checkpoint downregulates the activity of anti-tumor T cells synergistically with the PD-1 receptor. Another rationale for using it with anti-PD-1 ICIs is that tumors that are unresponsive to anti-PD-1 ICIs tend to have an increased level of TIM-3, suggesting its role in resistance to ICIs [85]. Therefore, a combination of ICIs that target TIM-3 and PD-1 is significantly more effective at protecting against tumors. Two anti-TIM-3 ICIs are now being tested in human clinical studies for a variety of solid cancers, including NSCLC (Table 1) [86].

#### 3.3.5. T Cell Ig and Immunoreceptor Tyrosine-Based Inhibitory Motif Domain (TIGIT)

T cell Ig and immunoreceptor tyrosine-based inhibitory motif domain (TIGIT) is a receptor that is homologous to CD28. It is expressed on the surface of NK, Treg and T cells [87]. It shares its ligands with CD226 in the same way CD28 and CTLA-4 share their ligands, but in this case the receptor CD226 is an immunostimulatory coreceptor whereas TIGIT is a coinhibitory receptor. It interacts with two ligands presented by antigen presenting cells, namely CD155 and CD112 [88]. TIGIT suppresses T cell activation, proliferation and signaling upon binding with its specific ligands [89]. In addition, its activation inhibits NK cell functions (Table 1) [90]. Specific interactions of TIGIT with CD155 ligand presented by DCs in murine cells have shown limited IL-12 production. There is a significant association between its expression and that of PD-1 receptors [91]. The anti-TIGIT antibody is currently being evaluated in human clinical trials alone or in combination with nivolumab in various solid locally advanced tumors [92].

**Table 1:** Summary of immune checkpoints for immune checkpoint inhibitor targeting.

Checkpoint Receptor	Checkpoint Ligand	Result of Interaction	Target ICI	References
PD-1	PD-L1	Repression of T cell-mediated tumor cell destruction by presenting tumor antigens via MHC	Anti-PD-1: pembrolizumab, nivolumab, cemiplimab Anti-PDL1: atezolizumab, avelumab, durvalumab	[93]
CTLA-4	CD80, CD86	Coinhibitory signals on T cells exert anti-tumor immune responses.	Ipilimumab	[94]
LAG-3	MHC	Terminating CD-4 associated T cell activity. Induction of DC maturation	Relatlimab, LAG525, Eftilagimod	[95]
VISTA	Unknown binding partners	Dampening the activity of tumor-specific T cells and inducing cytokine secretion	HMBD-002, JNJ-61610588, CA-170	[96]
HHLA2	TIMGD2	Co-stimulatory signal for T cell activation and cytokine release mediated by Akt dependent phosphorylation.	-	[97]
TIM-3	Gal-9	Apoptosis of T cells by inducing an influx of calcium into the cytoplasm	TSR-022	[98]
TIGIT	CD155, CD112	Inhibits the activation, proliferation, and signaling function of T cells Restricting the cytotoxic immune activity of NK cells	Etigilimab	[99]

Abbreviations: ICI: immune checkpoint inhibitors, PD-1: programmed cell death receptor-1, PD-L1: programmed cell death ligand-1, MHC: major histocompatibility complex, CTLA-4: cytotoxic T lymphocyte associated antigen, CD: cluster of differentiation, LAG-3: Lymphocyte activation gene-3, DC: dendritic cell, VISTA: V-Domain Ig Suppressor of T Cell Activation, HHLA2: Human Endogenous Retrovirus-H Long Terminal Repeat-Associating Protein 2, TIMGD2: transmembrane and immunoglobulin domain containing 2, Akt: Ak strain transforming, TIM-3: T Cell Immunoglobulin and Mucin-Containing Protein 3, Gal-9: Galectin-9, TIGIT: T Cell Ig and Immunoreceptor Tyrosine-Based Inhibitory Motif Doman.

## 4. Role of ICIs in the Treatment of Lung Cancer

Currently, FDA has approved seven ICIs: ipilimumab targeting CTLA-4 and pembrolizumab, nivolumab, durvalumab, atezolizumab, cemiplimab, and avelumab targeting PD-1/PD-L1.

### 4.1. ICIs as Monotherapy

ICIs when used as monotherapy for NSCLC patients have proved to be efficacious. When pembrolizumab was used as monotherapy in patients from the Eastern Cooperative Oncology Group (ECOG), performance status score < 2, PFS and OS were twice those of patients with low-performance status. Pembrolizumab monotherapy is the treatment of choice in patients showing high PD-L1 expression > 50%. The objective response rate (ORR) and disease control rate (DCR) in patients after pembrolizumab monotherapy were 30.9% and 41.5%, respectively, whereas when durvalumab was used as monotherapy in PD-L1, status > 25% showed a poor safety profile but progression free survival (PFS) and OS were found to be 4.3 and 7.9 months, respectively [100]. In another study, durvalumab was administered as monotherapy after chemoradiation in NSCLC. Median PFS was increased by 11.2 months in the durvalumab group compared to the placebo along with improvement in response rate and duration of response. The time for metastasis and death was longer in the durvalumab group with no increased toxicity [101]. When nivolumab was used as monotherapy, ORR was 28% in patients with positive PD-L1 status and 14% in patients with negative PD-L1 status, whereas median PFS and OS were 3.6 months and 19.4 months, respectively. The study also suggested a favorable toxicity profile for nivolumab [102]. In contrast to all these ICIs, atezolizumab has proved to be efficacious, even if only 1% of tumor cells show positive PD-L1 expression, when used as first-line treatment instead of chemotherapy. Median PFS was increased by 7.1 months in the subjects receiving atezolizumab compared to patients receiving chemotherapy. A comparatively lesser incidence of toxicity also took place in the atezolizumab group [103]. Ipilimumab is usually not used as monotherapy, but generally in combination with nivolumab. A study compared the effect of cemiplimab monotherapy to chemotherapy in NSCLC with at least >50% PD-L1 expression on tumor cells. Median PFS was significantly improved in the cemiplimab group by 2.5 months. Noteworthy improvements in PFS and OS were observed after cemiplimab therapy. Compared to chemotherapy, much fewer incidences of toxicity were seen with cemiplimab as first-line monotherapy in NSCLC [104]. Avelumab targeting PD-L1 is also proved to be efficacious in NSCLC. In a study targeting NSCLC, patients, regardless of their PD-L1 expression, were given avelumab as second-line post-platinum-based chemotherapy. Out these, 5.4% and 59.5% of patients achieved complete and partial responses, respectively, whereas disease stabilization was achieved in 29.7% of patients [105].

The ICIs have also been explored in SCLC patients as monotherapy. A group of SCLC patients was administered pembrolizumab monotherapy as second-line treatment in which PFS was 1.4 and OS was 9.6 months. This did not show any improved benefit when looking at the historical data. Thus, pembrolizumab monotherapy does not extend the PFS or OS in SCLC patients [106]. Durvalumab was administered as monotherapy in patients pretreated for SCLC as a second-line agent in a particular study. The ORR was found to be 9.5%, whereas the disease control rate (DCR) was 14.3%. Median PFS and OS were found to be 1.5 and 4.8 months. A noteworthy improvement in efficacy was reported with no major increase in toxicity profile [107]. A study was conducted on SCLC patients who relapsed after receiving platinum-based chemotherapy. These patients were given nivolumab monotherapy and the outcomes were compared to a group of patients receiving chemotherapy. The ORR in the nivolumab group was found to be 13.7%. Based on this, nivolumab monotherapy was approved in SCLC [108]. Atezolizumab has been evaluated as monotherapy in NSCLC but has not yet been much explored in SCLC. A clinical trial evaluating the safety and efficacy of atezolizumab in SCLC patients is currently ongoing. Considering currently available data for the same, the OS rate was 48% with an ORR of 19.5% [109]. Anti-CTLA-4 antibody ipilimumab is usually used along with other ICIs as combination therapy.

### 4.2. ICIs in Combination with Chemotherapy

The clinical safety and efficacy of ipilimumab in combination with chemotherapy versus placebo in NSCLC patients were evaluated. The median OS and PFS were 13.4 and 5.6 months, respectively. The results of this study were suggestive of the further requirement of research for combining ipilimumab with other agents. Its combination with chemotherapy does not show a significant increase in efficacy [110]. A significant improvement in efficacy was seen on administrating pembrolizumab with pemetrexed and platinum-based chemotherapy as opposed to chemotherapy plus placebo. There was a 19.8% increase in OS with positive PD-L1 status and the median PFS was 3.9 months higher in the pembrolizumab group compared to the placebo group. No significant toxicities were seen owing to pembrolizumab [111]. Pembrolizumab is mostly effective in patients with PD-L1 positive whereas other ICIs acting on PD-1 are not completely dependent on PD-L1 status. A combination of durvalumab with platinum-based chemotherapy in NSCLC patients regardless of their PD-L1 status was evaluated. The ORR was found to be 52.9% with no significant increase in toxicities [112]. Thus, the addition of durvalumab to chemotherapy might help in improving the disease outcomes compared to chemotherapy alone without added concerns for safety. A multi-arm study combined various drugs for the treatment of NSCLC. One arm used a combination of nivolumab with gemcitabine and cisplatin chemotherapy. In the group of patients receiving the combined treatment, 50% of patients achieved a partial response with a median PFS of 6.28 months along with no significant increase in dose limiting toxicity [113]. Similarly, nivolumab also shows significant improvement in the efficacy of chemotherapy when used in combination. As such, the combination of atezolizumab with platinum-based chemotherapy plus pemetrexed given as the first line in NSCLC patients was compared with a group of patients receiving chemotherapy alone. The median OS was increased by 8.6 months, whereas PFS was 8.3 months higher in the combination group. The safety of the combined treatment was also found to be tolerable [114]. A study was carried out on NSCLC patients receiving platinum doublet chemotherapy combined with cemiplimab to assess its safety and efficacy against a placebo. The median OS was seen to be improved by 8.9 months. Additionally, the combination group had a consistent safety profile and was linked to higher PFS, ORR, and longer duration of response [115]. Cemiplimab was proved to be efficacious with chemotherapy against a placebo. More detailed studies are required to prove its efficacy against chemotherapy alone. Patients of early-stage NSCLC that did not undergo surgical resection have shown positive results on the administration of chemotherapy and ICIs. In a group of patients, four doses of avelumab were co-administered with three neoadjuvant chemotherapy cycles pre-surgically. The results showed improved outcomes in efficacy and the combination proved to be safe with no increase in surgical complications [116].

**Figure 3:**
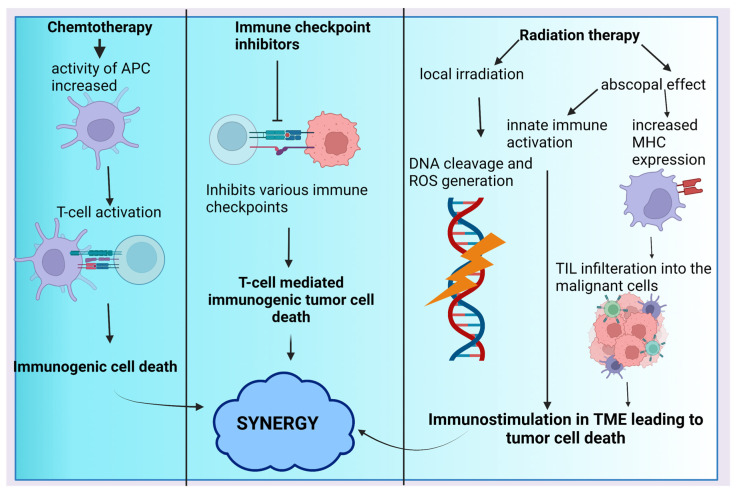
The rationale of combining ICIs with radiation and chemotherapy. This shows the influence of the immune system on the mechanism of conventional therapies. Though immune mediated tumor death is not the major mechanism for the antitumor effects of these conventional therapies, there is some amount of involvement of immune mediated mechanisms. This provides a rationale for combining immunotherapy with chemotherapy and radiation, as this might provide synergistic effects. Abbreviations: APC: antigen presenting cells, DNA: deoxy-ribo-nucleic acid, MHC: major histocompatibility complex, TIL: tumor infiltrating lymphocytes, TME: tumor microenvironment.

The combination of chemotherapy with ICIs is also being evaluated for clinical efficacy in SCLC. A study that combined paclitaxel with pembrolizumab showed moderate improvement in efficacy with a tolerable toxicity profile. The ORR in the SCLC patients with refractory disease was 23.1% out of which 3.8% of patients confirmed complete response and partial response was achieved in 19.2% of patients [117]. Another trial included pembrolizumab combined with all the commonly used chemotherapeutic regimens for SCLC to give a direction for future research using the combination that shows the best efficacy outcomes [118]. Durvalumab was combined with a platinum etoposide chemotherapy regimen. The OS in the combination group was increased by 25% compared to chemotherapy alone. The incidence of toxic effects was found to be the same in both groups. Thus, improved efficacy was noted with no increase in any risk of toxicity by combining durvalumab with chemotherapy [119]. A study assessing the efficacy of nivolumab combination with a cisplatin regimen with gemcitabine or pemetrexed was carried out. ORR and PFS for nivolumab combinations with the regimen containing gemcitabine is 47% and 71%, respectively, whereas for the pemetrexed-containing combination this was 47% and 38%, respectively [120]. Ipilimumab combined with carboplatin and etoposide chemotherapy in advanced-stage SCLC patients was evaluated. Median PFS in the subjects was found at 7.3 months. In addition, 72.4% of patients experienced a full recovery. Of the patients, 84.8% had an objective response. This combination might prove to be efficacious in SCLC patients [121]. A study evaluated the clinical benefit of combining first-line chemotherapy of carboplatin and etoposide of SCLC with atezolizumab. An increase of 2 months in median OS was observed in the patients given atezolizumab with chemotherapy compared to placebo along with an increase in PFS of 0.9 months. No new safety concerns arose during the administration of the combination [122]. These ICIs require further research in SCLC as well as more comprehensive data demonstrating their efficacy and safety. The rationale of adding ICIs to chemotherapy as well as radiotherapy has been depicted in Figure 3.

### 4.3. ICIs in Combination with Radiotherapy

Radiation therapy (RT) impacts its tumor by two mechanisms: the local irradiation effect and the abscopal effect. As such, RT can exert anti-tumor effects on distant regions that are not irradiated. This has recently been shown to be mediated by immune mechanisms. Thus, co-administration immunotherapy with RT helps to enhance its abscopal-mediated anti-tumor effect. Another rationale for combining RT with ICIs is that RT acts as a primer for various immunotherapeutic agents including ICIs by sensitizing the tumor cell to T cell activity [123]. Various randomized studies have shown the benefit of adding pembrolizumab to radiation therapy. An increase in PFS by 4.6 months and OD by 10.5 months was noted in patients receiving combination compared to pembrolizumab alone. Importantly, no additional safety concerns were seen in the combination group [124]. In patients with early-stage NSCLC, neoadjuvant durvalumab was combined with stereotactic radiotherapy. The groups receiving radiation in combination and the group receiving durvalumab monotherapy significantly differed in their key pathological responses. Thus, the addition of durvalumab to RT is well tolerated, safe, and also shows improved efficacy [125]. A retrospective analysis of nivolumab in patients eligible for hypo-fractionated radiation therapy as third-line palliative care was carried out. In comparison to patients receiving nivolumab alone, participants receiving the combination of nivolumab plus RT had higher yearly OS rates of 30.4% and PFS rates of 37.2%. There was no evident increase in acute toxicities by adding RT to nivolumab [126]. A study was conducted where patients were administered ipilimumab concurrently with RT. Three out of twenty-seven patients achieved clinically recognizable complete responses [127]. Two groups of patients were compared in a study, who were administered concurrent chemoradiation with one group receiving atezolizumab along with it. In the combo group, the average PFS was 13.2 months. The safety of the combination was proved with no added toxicities [128]. A combination of avelumab with specifically stereotactic ablative radiotherapy is currently being evaluated under the initial phases of clinical trials [123]. A comparative study was carried out to assess the effectiveness of adding cemiplimab to RT in patients with NSCLC who are supposed to receive radiation. The ORR was improved in the combination group by 21.8% whereas the disease control rate decreased when compared to the group receiving radiation alone. The safety of adding cemiplimab to radiation therapy was substantial [129].

Different ICIs have been used with RT to treat SCLC, and clinical trials are being conducted to evaluate the clinical efficacy and toxicity profile of these combinations. A phase II study was conducted in which nivolumab was given with stereotactic radiosurgery. The intracranial PFS was found to be 8 months whereas cumulative intracranial relapse was 17.4%. Extracranial PFS and OS were 2.9 and 14 months, respectively. RT along with nivolumab was found to be well tolerated [130]. Another clinical trial in progress is evaluating nivolumab, and ipilimumab along with stereotactic body RT. The results of this trial are not yet known. The other combination of tremelimumab and durvalumab is also being combined with RT. The PFS was 2.76 months, and the OS was 4.47 months. No major improvement in the efficacy of combining these ICIs with RT was noted. However, further detailed studies with a larger population are required to reach conclusive results [131]. When such further trials are carried out, more information about these ICIs and their function in the management of lung cancer alongside radiation therapy can be gained.

### 4.4. Combination of ICIs with Other Therapies

The utilization of ICI combinations in the management of lung cancer seems to be very important. A combination of dual-targeting ICIs can provide better efficacy compared to any of the ICIs used alone. For use as first-line therapy, the combination of ipilimumab and nivolumab alone or along with chemotherapy in NSCLC are most often used. Patients having positive PD-L1 status up to 1% had an OS of 17.1 months and a 40% 2-year OS rate. This combination did not have any additional safety concerns [132]. Evidence of combining durvalumab plus tremelimumab was provided by a study. The effectiveness of this combination was established in both PD-L1 positive as well as negative NSCLC patients; 23% of ORR was achieved on administering the combination, which is quite significant, as it was observed regardless of the PD-L1 status. The combination was also found to be tolerable [133]. These combinations of ICIs are termed a dual blockage as two checkpoints are targeted simultaneously. Looking at the results of the above trials, we can conclude that combining two ICIs can improve efficacy rather than a single agent, without any added toxicity issues.

The most common combination that is currently used in SCLC is ipilimumab plus nivolumab. The ORR of the combination according to the trial in which the combination was administered along with platinum-based chemotherapy was 23%, which is significantly higher than nivolumab alone. In addition, a substantial increase in adverse events with the combination is noted, but they are considered to be acceptable for recurrent SCLC [134]. This combination has also reported an increase in 9% ORR compared to nivolumab monotherapy [135]. Based on these results. triple ICI combinations are also evaluated in SCLC. Trials combining ipilimumab and nivolumab with an anti-GITR agonistic monoclonal antibody or an anti-OX40 agonistic antibody are being conducted. Another commonly used combination is durvalumab together with tremelimumab. The combination was given in SCLC patients and compared to the patients receiving durvalumab alone. This combination for SCLC has not yet been proven to be efficacious. Other than these, other combinations are also under clinical assessment for SCLC such as ipilimumab with pembrolizumab and tiragolumab plus atezolizumab. ICIs targeting other checkpoints such as LAG525 are also being assessed along with anti-PD-L1 agents.

Apart from this dual ICI therapy, they can also be used in combination with various other therapies for both NSCLC and SCLC. T cells and NK cells are known to have increased activity when exposed to IL-15. A super-agonist of IL-15 was combined with nivolumab to give 29% ORR with a tolerable toxicity profile. This also worked in patients resistant to PD-L1 targeted therapy [136]. This combination is also being assessed in SCLC considering the findings in NSCLC. Immune cells’ endosomes are known to contain the toll-like receptor-7 (TLR-7), which triggers the release of cytokines that promote inflammation. Combining TLR-7 agonists and ICIs can be beneficial. [137]. Other options for combinations include VEGF inhibitors, such as bevacizumab, which are being evaluated in combination with durvalumab [138]. Various tyrosine kinase inhibitors such as anlotinib, vorolanib, and cabozantinib are being evaluated with nivolumab [139]. Trilaciclib is a cyclin-dependent kinase 4/6 (CDK 4/6) inhibitor that is evaluated along with atezolizumab [140]. Apart from these agents, many pathways that have not yet been explored can be evaluated to develop novel agents that might be able to enhance the effectiveness of ICIs or could be useful in overcoming ICI resistance, producing a synergistic effect.

## 5. Clinical Trials of Various ICIs Targeting Immune Checkpoints

The clinical trials of prototypic immune checkpoints are also mentioned in Table 2. A landmark clinical trial (NCT01295827) evaluated pembrolizumab in various solid tumors including lung cancer. The drug was proved to be safe with no dose-limiting toxicities. The ORR was achieved in 21.6% patients [141]. As of now, various phase 2/3 clinical trials are ongoing to target PD-1/PD-L1 and CTLA-4.

Apart from the most commonly used checkpoint, PD-1 and CTLA-4, various other immune checkpoints have still not been clinically explored for use. The success of ICIs in lung cancer, such as pembrolizumab, nivolumab and ipilimumab, has pointed the research in the direction of working on checkpoint targets to develop novel targets and explore these. The expression of LAG on tumors was established by the TCGA, showing that its expression on various tumors has a direct effect on prognosis. Hui sun et al. conducted a study in a cohort of SCLC patients showing the LAG-3 expression in the tumor tissues and its association with PD-1 expression [142]. Thus, the development of a molecule targeting the LAG-3 checkpoint might prove to be efficacious. A clinical trial (NCT03625323) was conducted to assess the efficacy and tolerability of using a LAG-3 targeted ICI, Eftilagimod alpha, in NSCLC. The trial is still ongoing but initial results are promising, thus encouraging further research–48 patients were enrolled initially of which 35% had a stable illness, while 47% of patients had a partial response. There is not enough information to conclude on its safety and efficacy but promising outcomes are seen and further information will be available regarding its use in lung cancer therapy upon completion of the trial [143]. Jun Liu et al. suggested that mice with VISTA deficiency led to an acceleration in autoimmune diseases by stimulating T cells. Thus, it can be stipulated that VISTA along with PD-1 are required to keep a check on T-cell activation [144]. This is not just confined to the effect of VISTA on T lymphocytes but also has a large impact on the regulation of macrophages, which are known to promote tolerance and anti-inflammatory actions [145]. A clinical trial (NCT05082610) is currently ongoing to check the safety and tolerability of VISTA targeted monoclonal antibody, HMBD-002. The primary outcome of this trial is to find the tolerability, whereas efficacy outcomes will be measured as secondary outcomes. TIM-3 is also being considered as a potential target for ICIs in various malignancies including NSCLC. Extensive research is conducted in developing ICIs targeting novel checkpoints such as TIM-3. A meta-analysis was performed correlating the TIM-3 expression on tumors with disease prognosis such as poor overall survival, lymph node metastasis and PD-1 expression. When exploring TIM-3 related targets, it was found that it can be used along with PD-1 blockers as a dual blockage therapy. These dual blocking therapies are currently being explored through clinical trials [146]. The safety and effectiveness of using TSR-022, a monoclonal antibody against TIM-3, to treat cancer is being examined in a clinical trial (NCT03307785). A study conducted by Yaping Chen et al. concluded that, when tumor expressing TIGIT was exposed to TIGIT inhibitors in a murine model, it prolonged survival and delayed tumor progression. This can be used to consider TIGIT as a prospect for developing targeted ICIs [147].

Clinical research studies are being carried out on the drugs that target these unique checkpoints, as well as the initially mentioned prototypic checkpoints (Table 2).

**Table 2:** Clinical trials of ICIs targeting prototypic and alternative immune checkpoints.

Target Checkpoint	Trial No.	ICI Agent	Co-Administered with:	Phase of Trial	References
PD-1/PD-L1	NCT01295827 (KEYNOTE-001)	Pembrolizumab	-	Phase 1	[141]
	NCT02259621	Nivolumab	Carboplatin, Paclitaxel	Phase 2	[148]
	NCT04944173	Durvalumab	Stereotactic body radiotherapy	Phase 2	[149]
	NCT04513925 (SKYSCRAPER-03)	Atezolizumab	Tiragolumab, Durvalumab	Phase 3	[150]
	NCT02576574 (JAVELIN Lung 100)	Avelumab	Pemetrexed, Paclitaxel, Carboplatin, Gemcitabine	Phase 3	[151]
CTLA-4	NCT02477826 (CheckMate 227)	Ipilimumab	Nivolumab, Pemetrexed, Paclitaxel, Carboplatin, Gemcitabine	Phase 3	[152]
LAG-3	NCT02465060	Relatlimab	Nivolumab	Phase 2	[153]
	NCT02750514	Relatlimab	Nivolumab	Phase 2	[154]
	NCT03365791	LAG525 400 mg	PDR001	Phase 2	[155]
	NCT02460224	LAG525 0.3–10 mg/kg	PDR001	Phase 1/2	[156]
	NCT03625323	Eftilagimod alpha 30 mg	Pembrolizumab	Phase 2	[143]
VISTA	NCT05082610	HMBD-002	Pembrolizumab	Phase 1	[157]
	NCT02671955	JNJ-61610588	-	Phase 1	[158]
	NCT02812875	CA-170	-	Phase 1	[159]
TIM-3	NCT03307785	TSR-022 900 mg	Carboplatin + pemetrexed/nab-paclitaxel/paclitaxel	Phase 1	[160]
	NCT02817633	TSR-022	Nivolumab Docetaxel Cisplatin/carboplatin + pemetrexed	Phase 1	[161]
TIGIT	NCT05026606	Etigilimab	Nivolumab	Phase 2	
	NCT03119428	Etigilimab	Nivolumab	Phase 1	[162]

## 6. Limitations of ICI Therapy

Several studies have been conducted which showed better 5-year OS with the ICIs than with that of chemotherapy. According to a study comparing the effectiveness of DTX and nivolumab in increasing overall survival rates, DTX had a 5-year OS rate of 2.6% and nivolumab had a rate of 13.6%. The response rate after 5 years with nivolumab was 32.2% but no response was documented in the DTX arm. Chemotherapy over so many years could not improve OS by more than 10%.

### 6.1. Patients Harboring EGFR Mutation

Literature indicates a reduction in OS and PFS in a patient having EGFR mutations, specifically exon 19 deletions, when treated with ICIs [163]. Moreover, PD-L1 expression is reduced in EGFR mutant patients. Hence, the expression of PD-L1 can also be attributed to the activity of ICIs. As concluded by numerous studies, there is a direct relationship between the PD-L1 status of a tumor and the efficacy of ICIs [164]. Thus, it will be difficult to obtain the expected results in patients who have EGFR mutations.

### 6.2. Immune-Related Adverse Events (irAEs)

The targeted checkpoint, its dosage, and concomitant blocking all affect how often and how severe immune-related adverse events (irAEs) occur [165]. In contrast to ICIs that target PD-1/PD-L1, those that target the CTLA-4 checkpoint have a far higher chance of producing irAEs [166]. Higher toxicity is also seen on combining two ICIs, compared to when these are given as monotherapy [167]. These reactions are widely distributed, from mild to fatal in severity depending on various factors. The incidence rate of irAEs ranges as high as 66.4–75.1% in PD-1 targeted ICIs and up to 86.8% in CTLA-4 targeted ICIs [168]. Apart from this, a previous history of autoimmune diseases or interstitial pancreatitis acts as a separate risk factor for the occurrence of irAEs [169]. Generally, these irAEs affect various organ systems leading to dermatological, gastrointestinal, endocrine and rheumatological toxicities along with hepatitis and pneumonitis. First to be impacted are tissues high in lymphocytes, such as the skin and gut [170]. These are usually managed by discontinuing ICI therapy and initiating immunosuppressive therapy [171]. The major reason for the occurrence of irAEs is T cell diversity, cross-reactivity of self and tumor cells, and imbalance between T effector and Treg cells [172]. Apart from this, B cell-mediated mechanisms such as direct activation or autoantibody existence are also causative of these reactions [173]. The aberrant expressions of CTLA-4 in the pituitary gland, cytokine-mediated, or gut microbiome-mediated reactions are other mechanisms [174].

### 6.3. Coadministration of Steroids

Corticosteroids are usually administered in cancer patients owing to one indication or another, including symptomatic management and antiemetic for platinum-based chemotherapy. These steroids seem to counteract the activity of ICIs by antagonizing the immune response brought on by IL-12, as well as CD8+ T cells [175,176]. This also leads to increased activity of Tregs which also dampen the ICI activity. A few studies have also been reported showing the reduction in PFS due to the co-administration of steroids during ICI therapy [177]. On the contrary, these steroids are equally useful in the case of immune-mediated toxicities of the ICIs. Thus, the concurrent use of steroids during ICI treatment poses a challenge and is controversial [178]. Apart from the challenges to ICI therapy, another major concern remains that, if the tumor of the patient does not produce enough levels of PD-L1, it will not be detectable using these biomarkers [179]. Furthermore, lung cancer, being a heterogenous disease, is not limited to a few mutations. There are several neoantigens present which are not fixed during the disease. They keep evolving based on the TME and. thus, it becomes necessary to isolate and identify them [180].

## 7. Conclusions

Lung cancer is the most prevalent of its kind and is attributed for most cancer-related mortalities. Despite being currently managed with various chemotherapeutic regimens, satisfactory outcomes have not been achieved. Immunotherapy has been a recent prospect for the improvement of various outcomes in cancer. Until now, immunotherapy has always been looked at as an add-on therapy, thus masking its true efficacy. ICIs, a part of immunotherapy, have proved to exhibit significant therapeutic appeal and clinical rationale for lung cancer. Until now, limited ICIs were looked into and the research was limited to few checkpoints. ICIs currently used clinically target PD-1 and CTLA4 checkpoints. Various other immune checkpoints can be further studied, and drugs can be developed targeting these checkpoints. These novel checkpoints discussed above might be a step forward in the management of lung cancer. Despite the identification of other checkpoints, as discussed, the agents acting on them are not yet in clinical use. To inculcate these agents as a part of therapy, thorough strategical research is warranted.
